# (*E*)-4-Hydr­oxy-*N*′-(2-hydr­oxy-4-methoxy­benzyl­idene)benzohydrazide *N*,*N*-dimethyl­formamide solvate

**DOI:** 10.1107/S160053680804289X

**Published:** 2008-12-20

**Authors:** Nooraziah Mohd Lair, Hapipah Mohd Ali, Seik Weng Ng

**Affiliations:** a Department of Chemistry, University of Malaya, 50603 Kuala Lumpur, Malaysia

## Abstract

The Schiff base mol­ecule of the title compound, C_15_H_14_N_2_O_4_·C_3_H_7_NO, adopts a *trans* configuration with respect to the C=N double bond; the Schiff base itself is nearly planar (r.m.s. deviation 0.20 Å). The amido N atom is a hydrogen-bond donor to the dimethyl­formamide solvate mol­ecule. One of the hydr­oxy groups forms an intra­molecular hydrogen bond to the N atom of the C=N double bond, whereas the other forms an inter­molecular hydrogen bond to the carbonyl group.

## Related literature

For the corresponding monohydrate, see: Lair *et al.* (2009 ▶).
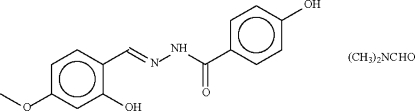


## Experimental

### 

#### Crystal data

C_15_H_14_N_2_O_4_·C_3_H_7_NO
*M* *_r_* = 359.38
Monoclinic, 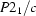

*a* = 11.8273 (2) Å
*b* = 7.8206 (2) Å
*c* = 19.4218 (3) Å
β = 103.674 (1)°
*V* = 1745.53 (6) Å^3^
*Z* = 4
Mo *K*α radiation
μ = 0.10 mm^−1^
*T* = 100 (2) K
0.30 × 0.25 × 0.15 mm

#### Data collection

Bruker SMART APEX diffractometer
Absorption correction: none
11825 measured reflections
4003 independent reflections
3303 reflections with *I* > 2σ(*I*)
*R*_int_ = 0.023

#### Refinement

*R*[*F* ^2^ > 2σ(*F* ^2^)] = 0.038
*wR*(*F* ^2^) = 0.104
*S* = 1.01
4003 reflections
240 parameters
H-atom parameters constrained
Δρ_max_ = 0.29 e Å^−3^
Δρ_min_ = −0.22 e Å^−3^

### 

Data collection: *APEX2* (Bruker, 2007 ▶); cell refinement: *SAINT* (Bruker, 2007 ▶); data reduction: *SAINT*; program(s) used to solve structure: *SHELXS97* (Sheldrick, 2008 ▶); program(s) used to refine structure: *SHELXL97* (Sheldrick, 2008 ▶); molecular graphics: *X-SEED* (Barbour, 2001 ▶); software used to prepare material for publication: *publCIF* (Westrip, 2009 ▶).

## Supplementary Material

Crystal structure: contains datablocks global, I. DOI: 10.1107/S160053680804289X/bt2837sup1.cif

Structure factors: contains datablocks I. DOI: 10.1107/S160053680804289X/bt2837Isup2.hkl

Additional supplementary materials:  crystallographic information; 3D view; checkCIF report

## Figures and Tables

**Table 1:** Hydrogen-bond geometry (Å, °)

*D*—H⋯*A*	*D*—H	H⋯*A*	*D*⋯*A*	*D*—H⋯*A*
O1—H1o⋯O2^i^	0.84	1.82	2.656 (1)	174
O3—H3o⋯N2	0.84	1.87	2.607 (1)	145
N1—H1n⋯O5	0.88	1.95	2.787 (1)	157

Symmetry code: (i) 
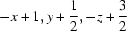
.

**Fig. 1.**
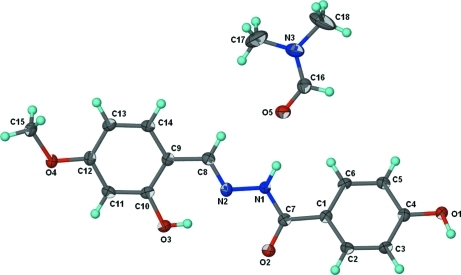
Displacement ellipsoid plot (Barbour, 2001) of the title compound at the 70% probability level. Hydrogen atoms are drawn as spheres of arbitrary radius.

**Fig. 2.**
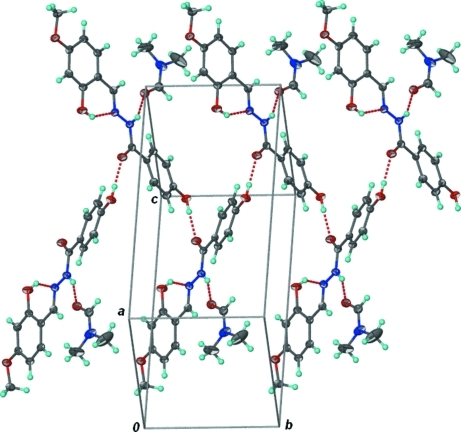
Packing diagram with hydrogen bonds drawn as dashed lines.

## supplementary crystallographic information

### Experimental

2-Hydroxy-3-methoxybenzaldehyde (0.30 g, 2 mmol) and 4-hydroxybenzohydrazide (0.30 g, 2 mmol) were heated in an ethanol-methanol mixture (50 ml) for 2 hours. The solvent was removed and the resulting compound recrystallized from DMF.

### Refinement

Hydrogen atoms were placed at calculated positions (C–H 0.95–0.98, N–H 0.88, O–H 0.84 Å) and were treated as riding on their parent carbon atoms, with *U*(H) set to 1.2–1.5 times *U*_eq_(*C*,*N*,*O*).

### Crystal data

**Table:**

C_15_H_14_N_2_O_4_·C_3_H_7_NO	*F*(000) = 760
*M**_r_* = 359.38	*D*_x_ = 1.368 Mg m^−^^3^
Monoclinic, *P*2_1_/*c*	Mo *K*α radiation, λ = 0.71073 Å
Hall symbol: -P 2ybc	Cell parameters from 4180 reflections
*a* = 11.8273 (2) Å	θ = 2.2–28.3°
*b* = 7.8206 (2) Å	µ = 0.10 mm^−^^1^
*c* = 19.4218 (3) Å	*T* = 100 K
β = 103.674 (1)°	Prism, colorless
*V* = 1745.53 (6) Å^3^	0.30 × 0.25 × 0.15 mm
*Z* = 4	

### Data collection

**Table:**

Bruker SMART APEX diffractometer	3303 reflections with *I* > 2σ(*I*)
Radiation source: fine-focus sealed tube	*R*_int_ = 0.023
graphite	θ_max_ = 27.5°, θ_min_ = 2.2°
ω scans	*h* = −15→14
11825 measured reflections	*k* = −10→8
4003 independent reflections	*l* = −25→25

### Refinement

**Table:**

Refinement on *F*^2^	Primary atom site location: structure-invariant direct methods
Least-squares matrix: full	Secondary atom site location: difference Fourier map
*R*[*F*^2^ > 2σ(*F*^2^)] = 0.038	Hydrogen site location: inferred from neighbouring sites
*wR*(*F*^2^) = 0.104	H-atom parameters constrained
*S* = 1.01	*w* = 1/[σ^2^(*F*_o_^2^) + (0.0515*P*)^2^ + 0.678*P*] where *P* = (*F*_o_^2^ + 2*F*_c_^2^)/3
4003 reflections	(Δ/σ)_max_ = 0.001
240 parameters	Δρ_max_ = 0.29 e Å^−^^3^
0 restraints	Δρ_min_ = −0.22 e Å^−^^3^

### Fractional atomic coordinates and isotropic or equivalent isotropic displacement parameters (Å^2^)

**Table:**

	*x*	*y*	*z*	*U*_iso_*/*U*_eq_	
O1	0.66809 (8)	0.74503 (12)	0.66928 (5)	0.0199 (2)	
H1O	0.6979	0.7676	0.7120	0.030*	
O2	0.22303 (8)	0.32357 (12)	0.69867 (4)	0.0211 (2)	
O3	−0.04810 (8)	0.04690 (13)	0.62396 (5)	0.0228 (2)	
H3O	0.0069	0.1161	0.6259	0.034*	
O4	−0.40063 (8)	−0.13116 (12)	0.47806 (5)	0.0200 (2)	
O5	0.18144 (8)	0.46309 (12)	0.44318 (5)	0.0227 (2)	
N1	0.17427 (9)	0.37007 (14)	0.58062 (5)	0.0170 (2)	
H1N	0.1888	0.4217	0.5434	0.020*	
N2	0.07635 (9)	0.27015 (14)	0.57472 (5)	0.0173 (2)	
N3	0.16184 (11)	0.57465 (17)	0.33359 (6)	0.0285 (3)	
C1	0.35639 (10)	0.48355 (16)	0.64886 (6)	0.0154 (2)	
C2	0.41943 (11)	0.53743 (17)	0.71534 (6)	0.0188 (3)	
H2	0.3901	0.5146	0.7559	0.023*	
C3	0.52370 (11)	0.62336 (17)	0.72326 (7)	0.0197 (3)	
H3	0.5656	0.6584	0.7690	0.024*	
C4	0.56747 (11)	0.65871 (16)	0.66420 (6)	0.0164 (3)	
C5	0.50566 (11)	0.60607 (16)	0.59728 (6)	0.0179 (3)	
H5	0.5351	0.6297	0.5568	0.022*	
C6	0.40136 (11)	0.51936 (16)	0.58983 (6)	0.0174 (3)	
H6	0.3597	0.4837	0.5441	0.021*	
C7	0.24710 (11)	0.38628 (16)	0.64518 (6)	0.0162 (2)	
C8	0.00648 (11)	0.25971 (16)	0.51315 (6)	0.0170 (3)	
H8	0.0228	0.3201	0.4742	0.020*	
C9	−0.09720 (10)	0.15539 (16)	0.50355 (6)	0.0158 (2)	
C10	−0.12163 (11)	0.05447 (16)	0.55883 (6)	0.0169 (3)	
C11	−0.22325 (11)	−0.04046 (16)	0.54772 (6)	0.0178 (3)	
H11	−0.2388	−0.1090	0.5848	0.021*	
C12	−0.30237 (11)	−0.03528 (16)	0.48223 (7)	0.0169 (3)	
C13	−0.28026 (11)	0.06116 (16)	0.42650 (7)	0.0183 (3)	
H13	−0.3341	0.0633	0.3817	0.022*	
C14	−0.17781 (11)	0.15370 (16)	0.43817 (6)	0.0173 (3)	
H14	−0.1617	0.2186	0.4002	0.021*	
C15	−0.49470 (11)	−0.10776 (18)	0.41743 (7)	0.0208 (3)	
H15A	−0.5643	−0.1651	0.4252	0.031*	
H15B	−0.4739	−0.1568	0.3757	0.031*	
H15C	−0.5105	0.0147	0.4098	0.031*	
C16	0.21144 (11)	0.56143 (17)	0.40187 (7)	0.0217 (3)	
H16	0.2757	0.6343	0.4204	0.026*	
C17	0.06495 (16)	0.4656 (3)	0.30112 (9)	0.0449 (5)	
H17A	0.0502	0.3839	0.3362	0.067*	
H17B	0.0836	0.4033	0.2614	0.067*	
H17C	−0.0045	0.5358	0.2837	0.067*	
C18	0.20628 (17)	0.6926 (3)	0.28847 (10)	0.0555 (6)	
H18A	0.2725	0.7558	0.3170	0.083*	
H18B	0.1448	0.7731	0.2665	0.083*	
H18C	0.2315	0.6283	0.2514	0.083*	

### Atomic displacement parameters (Å^2^)

**Table:**

	*U*^11^	*U*^22^	*U*^33^	*U*^12^	*U*^13^	*U*^23^
O1	0.0186 (4)	0.0259 (5)	0.0151 (4)	−0.0060 (4)	0.0037 (3)	−0.0012 (4)
O2	0.0185 (4)	0.0274 (5)	0.0168 (4)	−0.0028 (4)	0.0030 (3)	0.0036 (4)
O3	0.0202 (5)	0.0306 (6)	0.0156 (4)	−0.0057 (4)	0.0005 (4)	0.0032 (4)
O4	0.0167 (4)	0.0236 (5)	0.0188 (4)	−0.0051 (4)	0.0026 (4)	0.0005 (4)
O5	0.0268 (5)	0.0235 (5)	0.0173 (4)	−0.0020 (4)	0.0044 (4)	0.0008 (4)
N1	0.0158 (5)	0.0190 (5)	0.0155 (5)	−0.0034 (4)	0.0023 (4)	0.0013 (4)
N2	0.0149 (5)	0.0180 (5)	0.0191 (5)	−0.0012 (4)	0.0040 (4)	−0.0011 (4)
N3	0.0272 (6)	0.0369 (7)	0.0224 (6)	0.0071 (5)	0.0079 (5)	0.0088 (5)
C1	0.0155 (6)	0.0138 (6)	0.0166 (6)	0.0017 (5)	0.0027 (5)	0.0003 (4)
C2	0.0213 (6)	0.0210 (7)	0.0146 (6)	−0.0028 (5)	0.0054 (5)	−0.0002 (5)
C3	0.0224 (6)	0.0220 (7)	0.0140 (6)	−0.0037 (5)	0.0031 (5)	−0.0019 (5)
C4	0.0156 (6)	0.0152 (6)	0.0180 (6)	0.0003 (5)	0.0033 (5)	0.0004 (5)
C5	0.0196 (6)	0.0196 (6)	0.0155 (6)	0.0004 (5)	0.0058 (5)	0.0003 (5)
C6	0.0183 (6)	0.0188 (6)	0.0138 (6)	0.0009 (5)	0.0013 (5)	−0.0008 (5)
C7	0.0157 (6)	0.0159 (6)	0.0168 (6)	0.0021 (5)	0.0032 (5)	−0.0002 (5)
C8	0.0178 (6)	0.0168 (6)	0.0168 (6)	0.0008 (5)	0.0050 (5)	0.0004 (5)
C9	0.0146 (6)	0.0156 (6)	0.0173 (6)	0.0011 (5)	0.0041 (5)	−0.0013 (5)
C10	0.0167 (6)	0.0190 (6)	0.0145 (6)	0.0024 (5)	0.0028 (5)	−0.0008 (5)
C11	0.0195 (6)	0.0189 (6)	0.0161 (6)	0.0009 (5)	0.0064 (5)	0.0018 (5)
C12	0.0151 (6)	0.0160 (6)	0.0203 (6)	−0.0003 (5)	0.0055 (5)	−0.0027 (5)
C13	0.0173 (6)	0.0210 (7)	0.0156 (6)	0.0012 (5)	0.0016 (5)	−0.0002 (5)
C14	0.0189 (6)	0.0179 (6)	0.0151 (6)	0.0012 (5)	0.0042 (5)	0.0022 (5)
C15	0.0163 (6)	0.0252 (7)	0.0198 (6)	−0.0030 (5)	0.0022 (5)	−0.0017 (5)
C16	0.0197 (6)	0.0211 (7)	0.0239 (6)	0.0011 (5)	0.0045 (5)	0.0008 (5)
C17	0.0402 (10)	0.0651 (13)	0.0238 (8)	0.0006 (9)	−0.0040 (7)	−0.0066 (8)
C18	0.0496 (11)	0.0763 (15)	0.0460 (11)	0.0161 (10)	0.0217 (9)	0.0405 (10)

### Geometric parameters (Å, °)

**Table:**

O1—C4	1.3514 (15)	C5—H5	0.9500
O1—H1O	0.8400	C6—H6	0.9500
O2—C7	1.2412 (15)	C8—C9	1.4478 (17)
O3—C10	1.3565 (15)	C8—H8	0.9500
O3—H3O	0.8400	C9—C14	1.3962 (17)
O4—C12	1.3693 (15)	C9—C10	1.4162 (17)
O4—C15	1.4278 (15)	C10—C11	1.3855 (17)
O5—C16	1.2229 (16)	C11—C12	1.3901 (17)
N1—C7	1.3489 (15)	C11—H11	0.9500
N1—N2	1.3791 (14)	C12—C13	1.3938 (17)
N1—H1N	0.8800	C13—C14	1.3832 (17)
N2—C8	1.2855 (16)	C13—H13	0.9500
N3—C16	1.3212 (17)	C14—H14	0.9500
N3—C17	1.448 (2)	C15—H15A	0.9800
N3—C18	1.454 (2)	C15—H15B	0.9800
C1—C2	1.3948 (17)	C15—H15C	0.9800
C1—C6	1.4013 (17)	C16—H16	0.9500
C1—C7	1.4870 (17)	C17—H17A	0.9800
C2—C3	1.3808 (18)	C17—H17B	0.9800
C2—H2	0.9500	C17—H17C	0.9800
C3—C4	1.3931 (17)	C18—H18A	0.9800
C3—H3	0.9500	C18—H18B	0.9800
C4—C5	1.3943 (17)	C18—H18C	0.9800
C5—C6	1.3850 (17)		
			
C4—O1—H1O	109.5	O3—C10—C11	117.60 (11)
C10—O3—H3O	109.5	O3—C10—C9	122.04 (11)
C12—O4—C15	117.61 (10)	C11—C10—C9	120.36 (11)
C7—N1—N2	118.01 (10)	C10—C11—C12	119.89 (11)
C7—N1—H1N	121.0	C10—C11—H11	120.1
N2—N1—H1N	121.0	C12—C11—H11	120.1
C8—N2—N1	117.12 (10)	O4—C12—C11	114.61 (11)
C16—N3—C17	120.60 (13)	O4—C12—C13	124.26 (11)
C16—N3—C18	121.12 (14)	C11—C12—C13	121.13 (11)
C17—N3—C18	118.21 (14)	C14—C13—C12	118.28 (11)
C2—C1—C6	118.28 (11)	C14—C13—H13	120.9
C2—C1—C7	117.86 (11)	C12—C13—H13	120.9
C6—C1—C7	123.83 (11)	C13—C14—C9	122.50 (11)
C3—C2—C1	121.20 (11)	C13—C14—H14	118.7
C3—C2—H2	119.4	C9—C14—H14	118.7
C1—C2—H2	119.4	O4—C15—H15A	109.5
C2—C3—C4	120.06 (12)	O4—C15—H15B	109.5
C2—C3—H3	120.0	H15A—C15—H15B	109.5
C4—C3—H3	120.0	O4—C15—H15C	109.5
O1—C4—C3	122.10 (11)	H15A—C15—H15C	109.5
O1—C4—C5	118.30 (11)	H15B—C15—H15C	109.5
C3—C4—C5	119.60 (11)	O5—C16—N3	125.37 (13)
C6—C5—C4	119.98 (11)	O5—C16—H16	117.3
C6—C5—H5	120.0	N3—C16—H16	117.3
C4—C5—H5	120.0	N3—C17—H17A	109.5
C5—C6—C1	120.89 (11)	N3—C17—H17B	109.5
C5—C6—H6	119.6	H17A—C17—H17B	109.5
C1—C6—H6	119.6	N3—C17—H17C	109.5
O2—C7—N1	121.23 (11)	H17A—C17—H17C	109.5
O2—C7—C1	122.05 (11)	H17B—C17—H17C	109.5
N1—C7—C1	116.72 (11)	N3—C18—H18A	109.5
N2—C8—C9	119.62 (11)	N3—C18—H18B	109.5
N2—C8—H8	120.2	H18A—C18—H18B	109.5
C9—C8—H8	120.2	N3—C18—H18C	109.5
C14—C9—C10	117.80 (11)	H18A—C18—H18C	109.5
C14—C9—C8	119.86 (11)	H18B—C18—H18C	109.5
C10—C9—C8	122.34 (11)		
			
C7—N1—N2—C8	178.29 (11)	N2—C8—C9—C10	−4.09 (18)
C6—C1—C2—C3	−0.22 (19)	C14—C9—C10—O3	179.47 (11)
C7—C1—C2—C3	177.82 (12)	C8—C9—C10—O3	−1.25 (19)
C1—C2—C3—C4	0.4 (2)	C14—C9—C10—C11	−0.74 (18)
C2—C3—C4—O1	178.84 (12)	C8—C9—C10—C11	178.55 (11)
C2—C3—C4—C5	−0.3 (2)	O3—C10—C11—C12	179.08 (11)
O1—C4—C5—C6	−179.11 (11)	C9—C10—C11—C12	−0.72 (19)
C3—C4—C5—C6	0.07 (19)	C15—O4—C12—C11	168.75 (11)
C4—C5—C6—C1	0.10 (19)	C15—O4—C12—C13	−11.33 (17)
C2—C1—C6—C5	−0.02 (19)	C10—C11—C12—O4	−178.59 (11)
C7—C1—C6—C5	−177.94 (12)	C10—C11—C12—C13	1.49 (19)
N2—N1—C7—O2	−5.05 (18)	O4—C12—C13—C14	179.34 (11)
N2—N1—C7—C1	175.38 (10)	C11—C12—C13—C14	−0.74 (19)
C2—C1—C7—O2	−14.53 (18)	C12—C13—C14—C9	−0.79 (19)
C6—C1—C7—O2	163.40 (12)	C10—C9—C14—C13	1.52 (18)
C2—C1—C7—N1	165.03 (11)	C8—C9—C14—C13	−177.79 (12)
C6—C1—C7—N1	−17.04 (18)	C17—N3—C16—O5	−1.2 (2)
N1—N2—C8—C9	179.42 (11)	C18—N3—C16—O5	−178.22 (15)
N2—C8—C9—C14	175.18 (12)		

### Hydrogen-bond geometry (Å, °)

**Table:**

*D*—H···*A*	*D*—H	H···*A*	*D*···*A*	*D*—H···*A*
O1—H1o···O2^i^	0.84	1.82	2.656 (1)	174
O3—H3o···N2	0.84	1.87	2.607 (1)	145
N1—H1n···O5	0.88	1.95	2.787 (1)	157

Symmetry codes: (i) −*x*+1, *y*+1/2, −*z*+3/2.
